# Description of a new Asian Leaf Litter Toad of the genus *Leptobrachella* Smith, 1925 (Anura, Megophryidae) from southern Guizhou Province, China

**DOI:** 10.3897/BDJ.12.e113427

**Published:** 2024-01-09

**Authors:** Shize Li, Wei Li, Yanlin Cheng, Jing Liu, Gang Wei, Bin Wang

**Affiliations:** 1 Moutai Institute, Guizhou, China Moutai Institute Guizhou China; 2 Chengdu Institute of Biology, the Chinese Academy of Sciences, Chengdu, China Chengdu Institute of Biology, the Chinese Academy of Sciences Chengdu China; 3 Guiyang College, Guizhou, China Guiyang College Guizhou China

**Keywords:** Taxonomy, molecular phylogenetic analyses, morphology, new species

## Abstract

**Background:**

The Asian leaf litter toads of the genus *Leptobrachella* Smith, 1925 (Anura, Megophryidae) inhabit the forest floor and rocky streams in hilly evergreen forests and are widely distributed from southern China, west to north-eastern India and Myanmar, through mainland Indochina to Peninsular Malaysia and the Island of Borneo.

**New information:**

A new species of the Asian leaf litter toad genus *Leptobrachella* from Guizhou Province, China is described. Molecular phylogenetic analyses, based on mitochondrial 16S rRNA and COI genes and nuclear RAG1 gene sequences indicated that the new species is genetically divergent from its congeners. The new species could be distinguished from its congeners by a combination of the following characters: (1) body of medium size in males (SVL 31.9 – 32.9 mm); (2) distinct black spots present on flanks; (3) toes rudimentarily webbed, with wide lateral fringes; (4) skin on dorsum shagreened with fine tiny granules and short ridges; (5) heels overlapped when thighs are positioned at right angles to the body; (6) tibia-tarsal articulation reaching interior corner of the eye.

A new species of the Asian leaf litter toad genus *Leptobrachella* from Guizhou Province, China is described. Molecular phylogenetic analyses, based on mitochondrial 16S rRNA and COI genes and nuclear RAG1 gene sequences indicated that the new species is genetically divergent from its congeners. The new species could be distinguished from its congeners by a combination of the following characters: (1) body of medium size in males (SVL 31.9 – 32.9 mm); (2) distinct black spots present on flanks; (3) toes rudimentarily webbed, with wide lateral fringes; (4) skin on dorsum shagreened with fine tiny granules and short ridges; (5) heels overlapped when thighs are positioned at right angles to the body; (6) tibia-tarsal articulation reaching interior corner of the eye.

## Introduction

The Asian leaf litter toads of the genus *Leptobrachella* Smith, 1925 (Anura, Megophryidae) inhabit the forest floor and rocky streams in hilly evergreen forests and are widely distributed from southern China, west to north-eastern India and Myanmar, through mainland Indochina to Peninsular Malaysia and the Island of Borneo (Frost 2023). The species in the group had been classified into different genera, i.e. *Paramegophrys* Liu, 1964, *Carpophrys* Sichuan Biological Research Institute, 1977, *Leptolalax* Dubois, 1980, *Lalax* Delorme, Dubois, Grosjean & Ohler, 2006 and *Lalos* Dubois, Grosjean, Ohler, Adler & Zhao, 2010. Chen et al. (2018) suggested that the above genera were synonymised with *Leptobrachella*, based on large-scale molecular phylogenetic analyses. Currently, the genus contains 102 species, of which there are 40 species described in the last five years (Frost 2023). The species diversity in the genus was indicated to be much underestimated and many cryptic species have not been described till now (Chen et al. 2018), especially in some species group which were formerly recorded as widespread species, such as *L.oshanensis* complex (Nguyen et al. 2021, Shi et al. 2021) and more fieldwork and investigations should focus on them.

From 2022 to 2023, four specimens of *Leptobrachella* were collected from Dushan County, Guizhou Province, China. Morphologically, these specimens most closely resemble *L.dong*, but differ from *L.dong* and all other *Leptobrachella* from China and adjoining countries. To distinguish these specimens, we conducted phylogenetic analyses, based on mitochondrial DNA, nuclear DNA and morphological comparisons. All of the analyses consistently indicated that the specimens from Dushan County are a new taxon. Herein, we describe this taxon as a new species.

## Materials and methods

### Specimens

**Figure 1.**
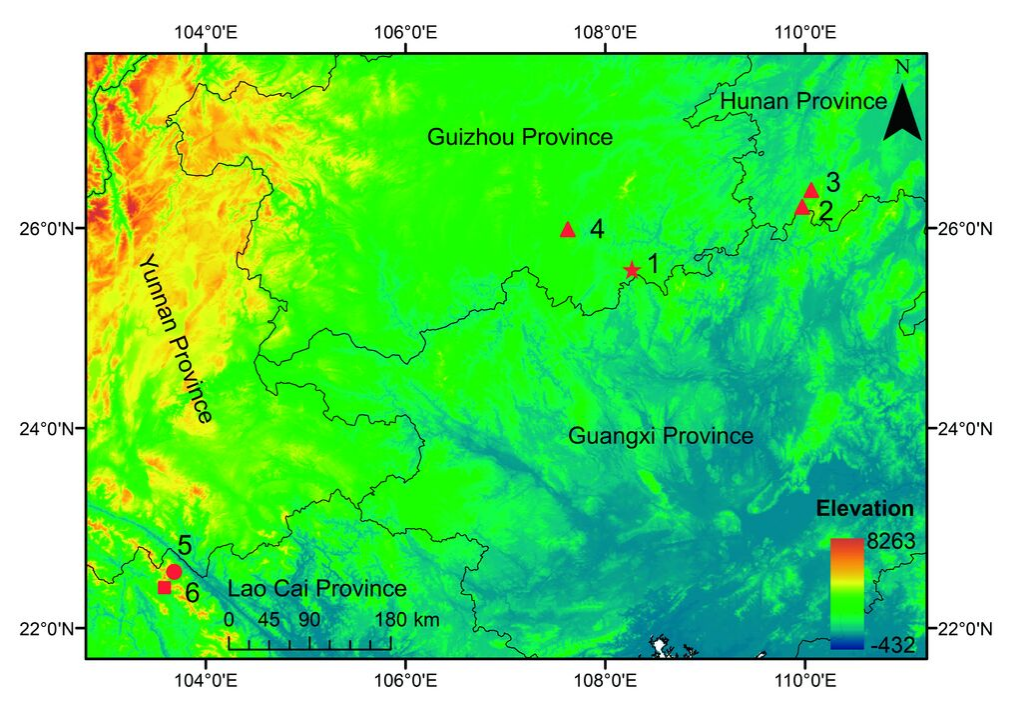
Sampling localities of *Leptobrachelladushanensis* sp. nov., *L.bourreti*, *L.dong* and *L.graminicola*. 1 Dushan County, the type locality of *Leptobrachelladushanensis* sp. nov.; 2-4 represent the sampling localities of *L.dong*; 2 Congjiang County, Guizhou Province; 3 Tongdao County, Hunan Province; 4 Suining County, Hunan Province, China; 5 represent the sampling locality of *L.bourreti* from Bat Xat District, Lao Cai, Vietnam; 6 the sampling locality of *L.graminicola* from Mount Pu Ta Leng, Lao Cai, Vietnam.

Four specimens of the undescribed species (Table 1 and Suppl. material 1) were collected from Dushan County, Guizhou Province, China (Fig. 1). After taking photographs, they were euthanized using isoflurane and then the specimens were fixed in 10% buffered formalin. Tissue samples were taken and preserved separately in 95% ethanol prior to fixation. Specimens were deposited in Moutai Institute (**MT**).

### Molecular phylogenetic analyses

Total DNA was extracted using a standard phenol-chloroform extraction protocol (Sambrook et al. 1989). The sequences of mitochondrial 16S rRNA (16S) and cytochrome oxidase subunit I (COI) genes and nuclear gene recombination activating gene 1 (RAG1) were amplified. For 16S, the primers P7 (5’-CGCCTGTTTACCAAAAACAT-3’) and P8 (5’-CCGGTCTGAACTCAGATCACGT-3’) were used following Simon et al. (1994), for COI, Chmf4 (5’-TYTCWACWAAYCAYAAAGAYATCGG-3’) and Chmr4 (5’-ACYTCRGGRTGRCCRAARAATCA-3’) were used following Che et al. (2012) and for RAG1, RAG1-F (AGCTGCAGYCARTACCAYAARATGTA) and RAG1-R (GCAAAGTTTCCGTTCATTCTCAT) were used following Mauro et al. (2004). The PCR amplification procedures are as follows: an initial denaturing step at 95°C for 4 min; 36 cycles of denaturing at 95°C for 30 s, annealing at 51°C (for 16S)/47°C (for COI)/57°C (for RAG1) for 30 s and extending at 72°C for 70 s. The fragments were sequenced on an ABI Prism 3730 automated DNA sequencer at Chengdu TSING KE Biological Technology Co. Ltd (Chengdu, China). New sequences were deposited in GenBank (for GenBank accession numbers, see Table 1).

For phylogenetic analyses, we downloaded the corresponding gene sequences for all related species in the genus *Leptobrachella* from GenBank, based on previous studies (Chen et al. 2018, Shi et al. 2021). Corresponding sequences of one *Leptobrachiumhuashen* Fei and Ye (2005) and one *Megophrysglandulosa* (Fei et al. 1990) were downloaded and used as outgroups.

Sequences were assembled and aligned using the Clustalw module in BioEdit v. 7.0.9.0 (Hall 1999) with default settings. Alignments were checked by eye and revised manually, if necessary. Phylogenetic analyses of mitochondrial DNA, the datasets of 16S and COI gene sequences were prepared using Maximum Likelihood (ML) and Bayesian Inference (BI) methods, implemented in PhyML v. 3.0 (Guindon et al. 2010) and MrBayes v. 3.12 (Ronquist and Huelsenbeck 2003), respectively. The best-fit nucleotide substitution models for the sequence super-matrices were selected in PartitionFinder 2.1.1 (Lanfear et al. 2012) using the Bayesian Information Criterion (BIC). As a result, the analysis suggested that the best model for 16S was GTR + R and, for the COI gene, it was TN93 + G + I. For the ML tree, branch supports were drawn from 10,000 non-parametric bootstrap replicates. In BI analyses, the parameters for each partition were unlinked and branch lengths were allowed to vary proportionately across partitions. Two runs each with four Markov chains were simultaneously run for 60 million generations with sampling every 1,000 generations. The first 25% trees were removed as the “burn-in” stage followed by calculations of Bayesian posterior probabilities and the 50% majority-rule consensus of the post burn-in trees sampled at stationarity. To detect the haplotype relationships and genetic isolation between the undescribed species and its related species on nuclear DNA, a haplotype network, based on RAG1 gene sequences, was constructed using the maximum parsimony method in TCS v.1.21 (Clement et al. 2000). Finally, genetic distance between *Leptobrachella* species, based on the uncorrected *p*-distance model, was estimated on 16S and COI genes using MEGA v. 6.06 (Tamura et al. 2013), respectively.

### Morphological comparisons

Morphologically, these specimens most closely resemble *L.dong.* To explore the morphological differences between the new taxon and *L.dong*, four specimens of the new taxon and 15 specimens of *L.dong* containing four specimens from Guizhou Province and 11 specimens from Hunan Province were measured. The terminology and methods followed Fei et al. (2005)and Liu et al. (2023). Measurements were made to the nearest 0.1 mm (Watters et al. 2016) with digital calipers. Fourteen morphometric characters of adult specimens were measured: eye diameter (ED, distance from the anterior corner to the posterior corner of the eye); foot length (FL, distance from tarsus to the tip of the fourth toe); head length (HDL, distance from the tip of the snout to the articulation of jaw); head width (HDW, greatest width between the left and right articulations of jaw); hind-limb length (HLL, distance from tip of fourth toe to vent); internasal distance (IND, minimum distance between the inner margins of the external nares); interorbital distance (IOD, minimum distance between the inner edges of the upper eyelids); length of lower arm and hand (LAL, distance from the elbow to the distal end of the Finger IV); snout length (SL, distance from the tip of the snout to the anterior corner of the eye); snout-vent lengt (SVL, distance from the tip of the snout to the vent); maximal tympanum diameter (TD); tibia length (TL, distance from knee to tarsus); tibia width (TW, the widest length of the tibia); upper eyelid width (UEW, greatest width of the upper eyelid margins measured perpendicular to the anterior-posterior axis).

In order to reduce the impact of allometry, the correct value from the ratio of each measurement to SVL was calculated and then log-transformed for the following morphometric analyses. One-way analysis of variance (ANOVA) was used to test the significance of differences on morphometric characters between the undescribed species and *L.dong* from different populations in males. The significance level was set at 0.05. To show the spatial distribution of different species on the morphometric characters, principal component analyses (PCA) were performed. All statistical analyses were performed using SPSS 21.0 (SPSS, Inc., Chicago, IL, USA).

The undescribed taxon was also compared with all other congeners of *Leptobrachella*, based on morphological characters. Comparative morphological data were obtained from literature (Table 2).

## Data resources

All the sequences in this study were retrieved from GenBank and the accession numbers of the newly-determined sequences in this study are shown in Table 1.

## Taxon treatments

### Leptobrachella dushanensis

Li, Li, Cheng, Liu, Wei, Wang sp. nov.

103BDF98-2D43-5B1E-B679-892A226BDAC6

50266F7E-6131-4795-9E8F-3ABD80E5471C

#### Materials

- **Type status:** Holotype. **Occurrence:** recordedBy: Jing Liu; sex: male; lifeStage: adult; occurrenceID: B8B9E666-2D12-5015-8398-05CB6F244FCD; **Taxon:** scientificName: *Leptobrachelladushanensis*; kingdom: Animalia; phylum: Chordata; class: Amphibia; order: Anura; family: Megophryidae; genus: Leptobrachella; **Location:** country: China; stateProvince: Guizhou; county: Dushan; decimalLatitude: 25.961719; decimalLongitude: 107.649194; **Identification:** identifiedBy: Shize Li; **Event:** eventDate: 2022-04-09; **Record Level:** institutionID: MT DS20220409002; collectionID: DS20220409002
- **Type status:** Paratype. **Occurrence:** recordedBy: Jing Liu and Wei Li; sex: 3 males; lifeStage: adult; occurrenceID: 83945396-6D45-5955-875D-54024A94F66A; **Taxon:** scientificName: *Leptobrachelladushanensis*; kingdom: Animalia; phylum: Chordata; class: Amphibia; order: Anura; family: Megophryidae; genus: Leptobrachella; **Location:** country: China; stateProvince: Guizhou; county: Dushan; decimalLatitude: 25.961719; decimalLongitude: 107.649194; **Identification:** identifiedBy: Shize Li; **Record Level:** type: Event; institutionID: MT DS20220409001, MT DS20230310001 and MT DS20230310002; collectionID: DS20220409001, DS20230310001 and DS20230310002

#### Diagnosis

*Leptobrachelladushanensis* sp. nov. is assigned to the genus *Leptobrachella*, based on molecular data and the following morphological characters: medium size, rounded finger tips, the presence of two elevated inner palmar tubercle not continuous to the thumb, presence of macroglands on body (including supra-axillary, pectoral and femoral glands), vomerine teeth absent, tubercles on eyelids and anterior tip of snout with vertical white bar (Dubois 1983; Fei et al. 2009).

*Leptobrachelladushanensis* sp. nov. could be distinguished from its congeners by a combination of the following characters: body of medium size in males (SVL 31.9 – 32.9 mm); distinct black spots present on flanks; toes rudimentarily webbed, with wide lateral fringes; skin on dorsum shagreened with fine tiny granules and short ridges; heels overlapped when thighs are positioned at right angles to the body; tibia-tarsal articulation reaching interior corner of the eye.

##### Description of holotype

Adult male. SVL in 33.2 mm. Head length slightly wider than head width (HDL/HDW = 1.04); snout sharply rounded in dorsal view, projecting slightly beyond margin of the lower jaw; nostril closer to snout than eye; loreal region oblique; canthus rostralis indistinct; eyes large and convex (ED/HDL = 0.37), slightly shorter than snout length (ED/SL = 0.93), pupil vertical; tympanum distinct, rounded, tympanum diameter smaller than eye (TD/ED = 0.43), upper margin of tympanum in contact with supratympanic ridge; vomerine teeth absent; tongue notched behind; supratympanic ridge distinct, extending from posterior corner of eye to supra-axillary gland.

Fore-limb relatively long (LAL / SVL = 0.45), fingers long and slender (ML/SVL = 0.25), without webbing, lateral fringes on fingers narrow; relative finger lengths II < I < IV < III; tips of fingers rounded and slightly swollen; subarticular tubercles absent on fingers, inner metacarpal tubercle large and rounded, separated from the smaller, round outer metacarpal; supra-axillary glands oval.

Hind-limb relatively long (HLL/SVL = 1.56), heels overlapping when the tibiae perpendicular to the body axis; tibio-tarsal articulation of adpressed limb reaching interior corner of the eye, tibia length about half of snout-vent length (TL/SVL = 0.49); relative toe length: I < II < III < V < IV; toe tips rounded and slightly swollen; rudimentary webbing present between all five toes; wide lateral fringes present on all toes; subarticular tubercles indistinct on the base of toe; inner metatarsal tubercle oval and distinct, outer metatarsal tubercle absent.

Skin on dorsum shagreened with fine tiny granules and short ridges; supra-axillary glands long, oval, close to the armpit; pectoral gland indistinct; round femoral glands present and protuberant on rear of thigh, closer to knee than to vent; femoral adipose glands distinct, attached to inner side of skin on posterior ventral surface of thigh; ventral skin smooth; ventrolateral glands distinctly visible and raised, forming an incomplete line.

##### Colouration of holotype in life

**Figure 2.**
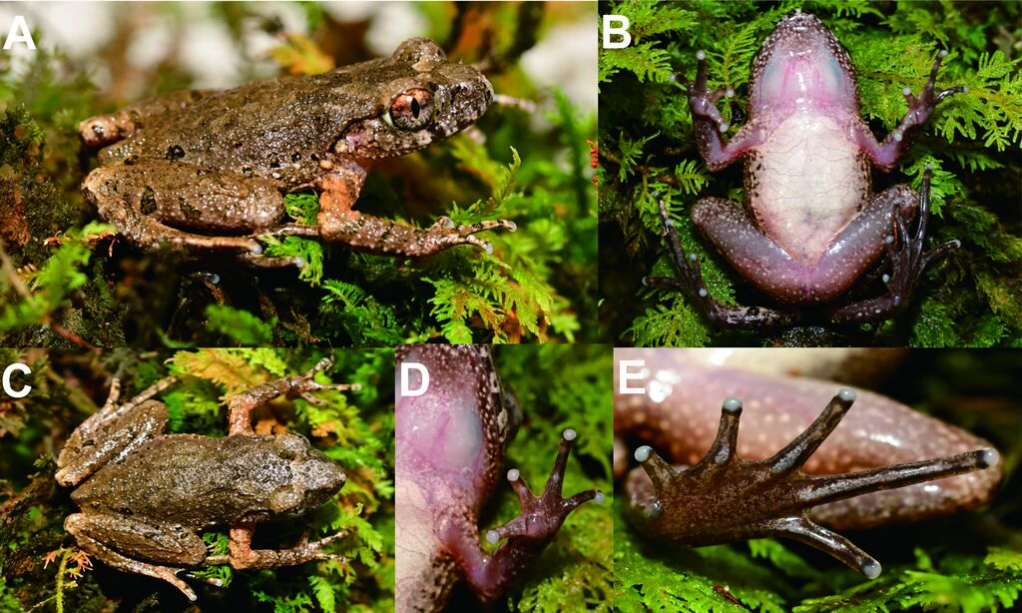
Photos of the holotype CIB DS20220409002 *Leptobrachelladushanensis* sp. nov. in life. **A** dorsolateral view; **B** ventral view; **C** dorsal view; **D** ventral view of hand **E** ventral view of foot.

In life, dorsal surface of head and trunk earth brown, with a distinct reverse-triangle taupe markings between eyes connecting to a taupe W-shaped marking between axillae that are fringed with greyish-white colour; very distinct, light brown markings between the nostrils; bicoloured iris, with the upper 1/3 of the iris being copper-orange and the lower 2/3 a light silvery-grey and a dark blotch under the eye; sparse, small, light brown granules and small dark brown patches present on the dorsum of the limbs; elbow to upper arm distinctly greyish-brown in colour on the dorsum; three transverse black bars present on dorsal surface of lower arm; distinct dark blotches on ﬂanks from groin to axilla, longitudinally in two rows; ventral surfaces light coloured, throat and ventral arms pinkish, chest and belly cream-white and ﬂanks of ventral with several granules and brown spots; ventral hind-limbs pinkish with sparse white glands (Fig. 2).

##### Preserved holotype colouration

**Figure 3.**
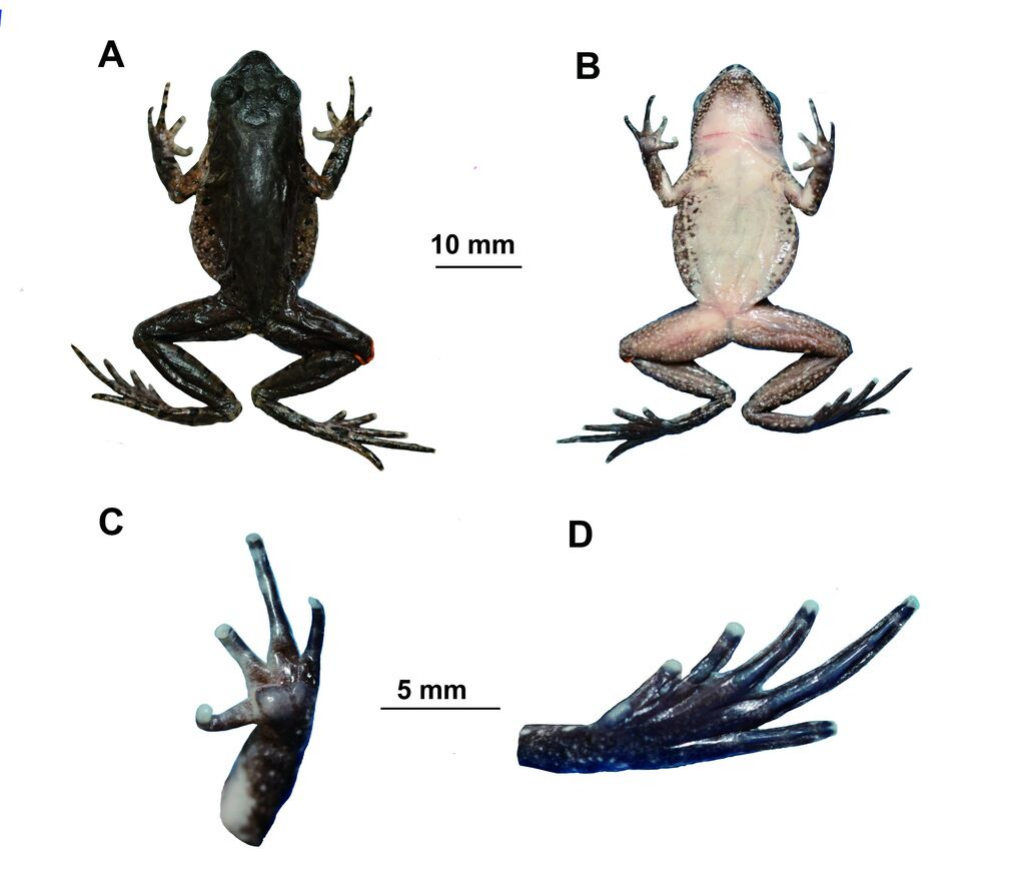
The holotype specimen MT DS20220409002 *Leptobrachelladushanensis* sp. nov. **A** dorsal view; **B** ventral view; **C** ventral view of hand; **D** ventral view of foot.

Dorsum of body and limbs fade to brown; transverse bars on limbs become more distinct. Ventral surface of body and limbs fade to cream-white. Supra-axillary, femoral and pectoral glands fade to cream-yellow (Fig. 3).

##### Variations

**Figure 4.**
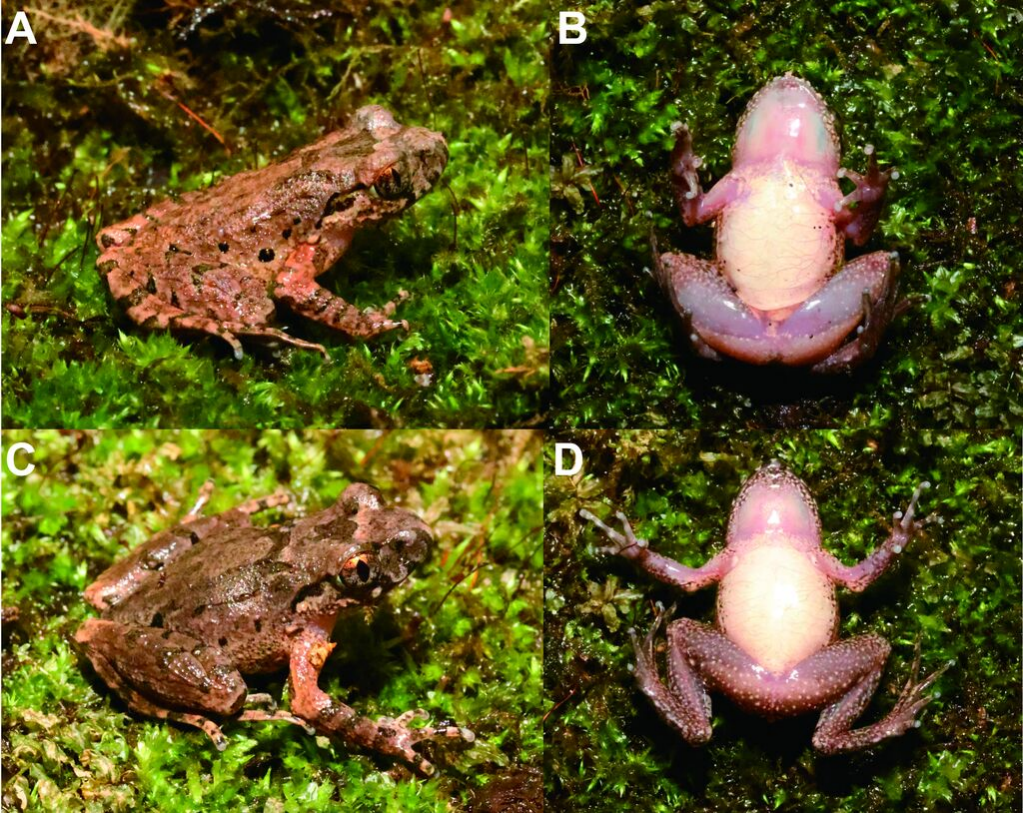
Colour variation in *Leptobrachelladushanensis* sp. nov. **A** dorsal view of the male specimen MT DS20230310001; **B** ventral view of the male specimen MT DS20230310001; **C** dorsal view of the male specimen MT DS20230310002; **D** ventral view of the female specimen MT DS20230310002.

Measurements of adult specimens are presented in Suppl. material 1 and Table 3, respectively. In MT DS20230310001, dorsal surface of head and trunk reddish-brown (Fig. 4A) and femoral adipose glands more obvious, ventral skin of thigh smooth (Fig. 4B); in MT DS20230310002, the black bars on dorsum more obvious (Fig. 4C) and glands on ventral surface of hind-limbs more dense (Fig. 4D).

##### Comparisons

Compared with the 26 known congeners occurring south of the Isthmus of Kra, *Leptobrachelladushanensis* sp. nov. could be distinguished from them by several characters. By having supra-axillary and ventrolateral glands, the new species differs from *L.arayai*, *L.dringi*, *L.fritinniens*, *L.gracilis*, *L.hamidi*, *L.heteropus*, *L.kajangensis*, *L.kecil*, *L.marmorata*, *L.maura*, *L.melanoleuca*, *L.picta*, *L.platycephala*, *L.sabahmontana* and *L.sola* (vs. absent in the latter); by having rounded fingertips and moderate body size (31.9 – 32.9 mm in four adult males), the new species differs from the following species with pointed fingertips and smaller body size: *L.baluensis* (14.9–15.9 mm in males), *L.bondangensis* (17.8 mm in male), *L.brevicrus* (17.1–17.8 mm in males), *L.fusca* (16.3 mm in male), *L.itiokai* (15.2–16.7 mm in males), *L.juliandringi* (17.0–17.2 mm in males), *L.mjobergi* (15.7–19.0 mm in males), *L.natunae* (17.6 mm in one adult male), *L.palmata* (14.4–16.8 mm in males), *L.parva* (15.0–16.9 mm in males) and *L.serasanae* (16.9 mm in female).

*Leptobrachelladushanensis* sp. nov. could also be identified from 76 known *Leptobrachella* species occurring north of the Isthmus of Kra by some characters (Suppl. material 2).

By having medium size of body (SVL 31.9–32.9 mm in males), *Leptobrachelladushanensis* sp. nov. differs from the the smaller in males *L.aerea* (25.1–28.9 mm), *L.alpina* (24.0–26.4 mm), *L.applebyi* (19.6–22.3 mm), *L.ardens* (21.3–24.7 mm), *L.aspera* (22.4 mm), *L.bashaensis* (22.9–25.6 mm), *L.bidoupensis* (23.6–24.6), *L.bijie* (29.0–30.4), *L.crocea* (22.2–27.3 mm), *L.dorsospina* (28.7–30.5 mm), *L.feii* (21.5–22.8 mm), *L.firthi* (26.4–29.2 mm), *L.flaviglandulosa* (23.0–27.0 mm), *L.fuliginosa* (28.2–30.0 mm), *L.graminicola* (23.1–24.6 mm) , *L.isos* (23.7–27.9 mm), *L.khasiorum* (24.5–27.3 mm), *L.lateralis* (26.9–28.3 mm), *L.laui* (24.8–26.7 mm), *L.liui* (24.8–26.7 mm), *L.macrops* (28.0–29.3 mm), *L.maculosa* (24.2–26.6 mm), *L.mangshanensis* (22.22–27.76 mm), *L.maura* (26.1 mm), *L.melica* (19.5–22.8 mm), *L.murphyi* (23.2–24.9 mm), *L.niveimontis* (22.5–23.6 mm), *L.pallida* (24.5–27.7 mm, *L.petrops* (23.6–27.6 mm), *L.pingbianensis* (28 mm), *L.pluvialis* (21.3–22.3 mm), *L.puhoatensis* (24.2–28.1 mm), *L.purpuraventra* (27.3–29.8 mm), *L.purpurus* (25.0–27.5 mm), *L.rowleyae* (23.4–25.4 mm), *L.shangsiensis* (24.9–29.4 mm), *L.shiwandashanensis* (26.8–29.7 mm), *L.shimentaina* (26.4–28.9 mm), *L.sinorensis* (26.6–27.1 mm), *L.suiyangensis* (28.7–29.7 mm), *L.tadungensis* (23.3–28.2 mm), *L.tengchongensis* (23.9–26.0 mm), *L.tuberosa* (24.4–29.5 mm), *L.ventripunctata* (23.7–27.7 mm), *L.verrucosa* (23.2–25.9 mm), L.wuhuangmontis (25.6–30.0 mm), *L.wumingensis* (26.0–26.7 mm), *L.yingjiangensis* (25.7–27.6 mm) and *L.yunkaiensis* (25.9–29.3 mm); differs from the larger in males *L.nahangensis* (40.8 mm), *L.platycephala* (35.1 mm), *L.sungi* (48.3–52.7 mm in males) and *L.zhangyapingi* (45.8–52.5 mm).

By having black spots on ﬂanks, *Leptobrachelladushanensis* sp. nov. differs from *L.aerea*, *L.botsfordi*, *L.crocea*, *L.eos*, *L.firthi*, *L.isos*, *L.pallida*, *L.petrops*, *L.tuberosa* and *L.zhangyapingi* (vs. lacking distinct black spots on the ﬂanks in the latter).

By having rudimentary webbing, *Leptobrachelladushanensis* sp. nov. differs from *L.ardens*, *L.jinshaensis*, *L.kalonensis*, *L.maculosa*, *L.oshanensis*, *L.pallida*, *L.petrops*, *L.rowleyae*, *L.shiwandashanensis* and *L.tadungensis* (vs. absent webbing in the latter).

By having wide fringes on toes, *Leptobrachelladushanensis* sp. nov. differs from *L.applebyi*, *L.ardens*, *L.aspera*, *L.bashaensis*, *L.bidoupensis*, *L.bijie*, *L.botsfordi*, *L.bourreti*, *L.chishuiensis*, *L.crocea*, *L.damingshanensis*, *L.dorsospina*, *L.feii, L.flaviglandulosa*, *L.fuliginosa*, *L.jinshaensis*, *L.kalonensis*, *L.korifi*, *L.lateralis*, *L.macrops*, *L.maculosa*, *L.mangshanensis*, *L.melica*, *L.minima*, *L.nahangensis*, *L.namdongensis*, *L.niveimontis*, *L.nyx*, *L.oshanensis*, *L.pallida*, *L.pelodytoides*, *L.petrops*, *L.phiaoacensis, L.phiadenensis*,

*L.pluvialis*, *L.puhoatensis*, *L.purpuraventra*, *L.pyrrhops*, *L.rowleyae*, *L.shangsiensis*, *L.sinorensis*, *L.shiwandashanensis*, *L.sungi*, *L.tengchongensis*, *L.tuberosa*, *L.ventripunctata*, *L.wuhuangmontis*, *L.wulingensis, L.wumingensis, L.yeae* and *L.yunyangensis* (vs. fringes on toes narrow or absent in the latter).

By having dorsal surface shagreened with fine tubercles, *Leptobrachelladushanensis* sp. nov. differs from *L.applebyi*, *L.bidoupensis*, *L.kalonensis*, *L.melica*, *L.minima*, *L.nahangensis*, *L.shangsiensis* and *L.tadungensis*, all of which have the dorsum smooth and *L.bourreti* (dorsum smooth with small warts), *L.fuliginosa* (dorsum smooth with fine tubercles), *L.liui* (dorsum with round tubercles), *L.macrops* (dorsum roughly granular with large tubercles), *L.maoershanensis* (dorsum shagreened with tubercles), *L.minima* (dorsum smooth), *L.neangi* (dorsum with small, irregular bumps and ridges), *L.nyx* (dorsum with round tubercles), *L.nokrekensis* (dorsum tubercles and longitudinal folds), *L.pelodytoides* (dorsum with small, smooth warts), *L.tamdil* (dorsum weakly tuberculate, with low, oval tubercles), *L.tuberosa* (dorsum highly tuberculate), *L.yunkaiensis* (dorsum with raised warts) and *L.wuhuangmontis* (dorsum rough with conical tubercles).

In mitochondrial DNA trees, *Leptobrachelladushanensis* sp. nov. and *L.dong* clustered into one clade, being sisters. The new species differs from *L.dong* by the following characters: head length slightly wider than head width (vs. head width slightly wider than head length); males with internal single subgular vocal sac (vs. a pair of subgular internal vocal sacs); tibiotarsal articulation reaching to anterior edge of eye (vs. reaching to middle of eye).

##### Secondary sexual characteristics

Adult males with internal single subgular vocal sac, femoral adipose glands present on posterior surface of thigh and tiny transparent spines on chest during breeding season. Nuptial pads and spines absent on males.

#### Etymology

This specifc name “Dushan” refers to the distribution of this species in Dushan County, Guizhou Province, China. We suggest its English common name “Dushan leaf litter toad” and Chinese name “Dushan Zhang Tu Chan (独山掌突蟾)”.

#### Distribution

*Leptobrachelladushanensis* sp. nov. was only found in Dushan County, Guizhou Province, China. Elevations recorded range from 1000 m to 1200 m.

#### Ecology

**Figure 5.**
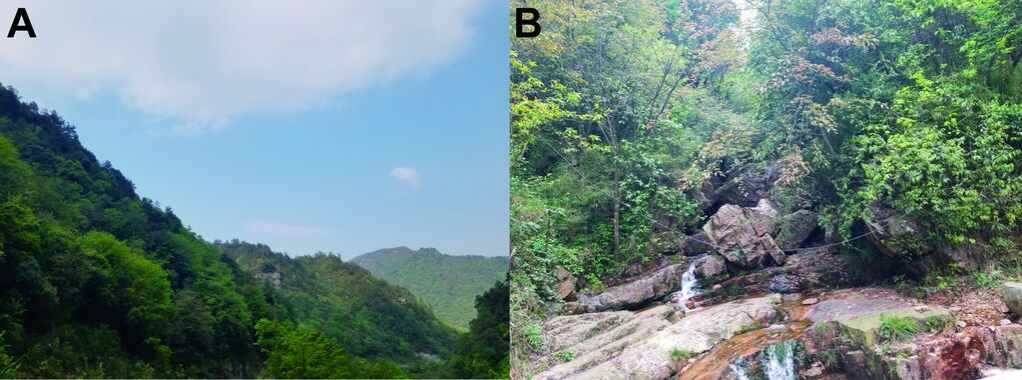
Habitats of *Leptobrachelladushanensis* sp. nov. **A** landscape of the type locality Dushan County, Guizhou Province, China; **B** a mountain stream in the type locality.

*Leptobrachelladushanensis* sp. nov. was found under stones in fast-flowing mountain streams surrounded by evergreen broadleaf forest (Fig. 5) and we did not find eggs, nor tadpoles or females. Based on our surveys, we speculate that the breeding season is probably in late March.

## Analysis

### Phylogenetic analyses

**Figure 6.**
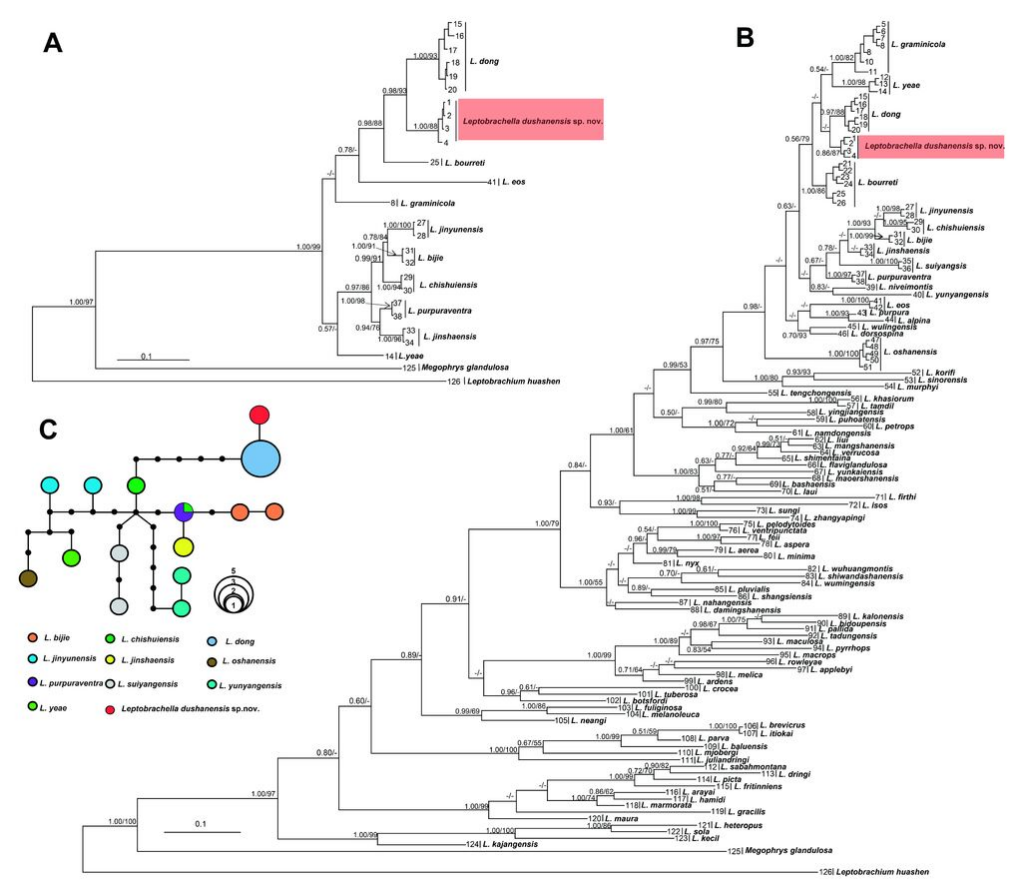
Phylogenetic trees of the genus *Leptobrachella* and a haplotype network constructed, based on RAG1 gene sequences. **A** Bayesian inference (BI) tree, based on mitochondrial COI gene; **B** Bayesian Inference (BI) tree, based on mitochondrial 16S gene; **C** the haplotype network constructed, based on RAG1 gene sequences. In this phylogenetic tree, Bayesian posterior probabilities (BPP) from BI analyses/bootstrap supports (BS) from ML analyses are listed beside the nodes. The symbol “-” represents a value below 0.50/50. For information of samples 1–126, refer to Table 1.

Aligned sequence matrix of 16S, COI and RAG1 genes contained 519, 615 and 831 bps respectively. ML and BI analyses, based on 16S and COI, resulted in essentially identical topologies. All samples of the undescribed species were clustered into one clade being deeply clustered into the *Leptobrachella* clade and being sister to *L.dong* (Fig. 6A and B). Only one haplotype was found for all samples of the undescribed species in RAG1 gene and there was no common haplotype between the undescribed species and its related species (Fig. 6C). The smallest pairwise genetic divergence between the undescribed species and its close congener *L.dong* from Guizhou population and Hunan population were both 1.6% on 16S (Suppl. material 3) and, on COI, were 7.3% and 7.4%, respectively (Suppl. material 4), which was higher than the divergence between the populations of *L.dong* (0.3% on 16S and 1.6% on COI) and also higher or at the same level with those amongst many pairs of congeners, such as *L.bijie* and *L.jinyunensis* (1.6% on 16S), *L.bijie* and *L.jinyunensis* was 4.4%, *L.bijie* and *L.chishuiensis* was 4.1%, *L.chishuiensis* and *L.jinshaensis* 5.8%, *L.jinshaensis* and *L.purpuraventra* was 4.0% on COI (Suppl. materials 3, 4).

### Morphological analyses

**Figure 7.**
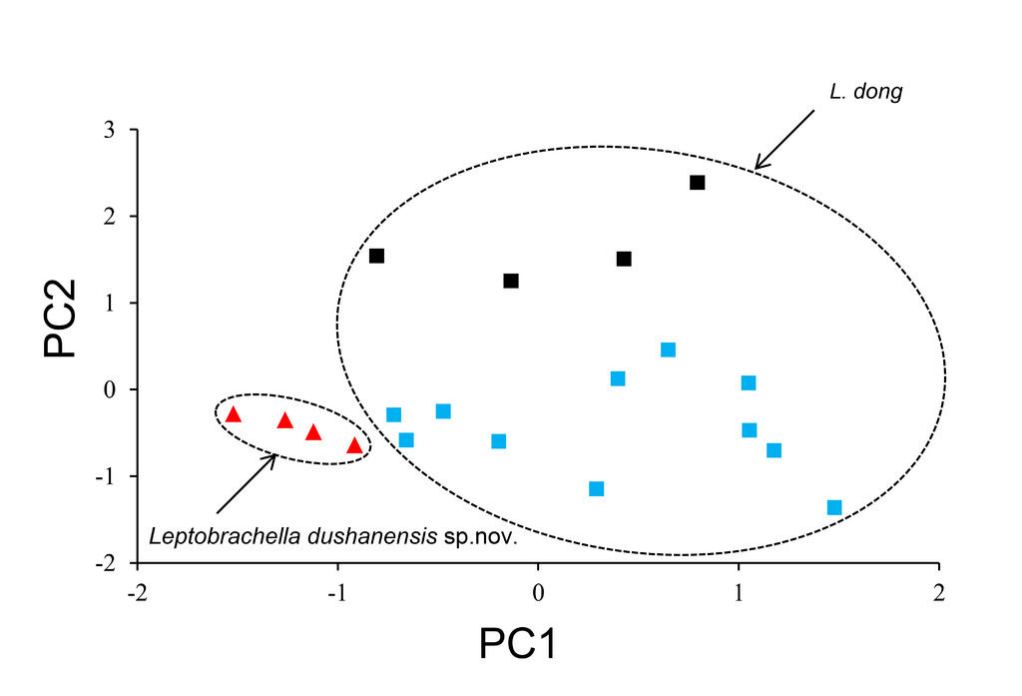
Plots of principal component analyses of *Leptobrachelladushanensis* sp. nov. and *L.dong* in males. PC1, the first principal component; PC2, the second principal component. Different species were denoted as different shapes, the red triangle represents *Leptobrachelladushanensis* sp. nov., black square represents *L.dong* from Guizhou Province and blue square represents *L.dong* from Hunan Province.

The results of one-way ANOVA showed that males of the undescribed species differed significantly from *L.dong* from both Guizhou and Hunan populations, based on several morphometric characters. From Guizhou population, the undescribed species was larger in SVL, HDW and HLL and shorter in SL, IND, ED and TYD; from Hunan population, the undescribed species was larger in HDL and shorter in HDW, SL, IND, IOD, ED and LAL (all *p*-values < 0.05; Table 3). In PCA for males, the total variation of the first two principal components was 57.52% and, on the two-dimensional plots of PC1 vs. PC2, the undescribed species could be separated from the specimens of *L.dong* both from Guizhou and Hunan populations (Fig. 7).

More detailed descriptions of results from morphological comparisons between the undescribed species and its congeners were presented in the Taxon Treatment section for describing the new species.

As a result, molecular and morphological results supported that our specimens from Guizhou Province, China were a new taxon.

## Discussion

The Asian leaf litter toads of *Leptobrachella* have low vagility and an exclusive association with montane forests and their populations are often highly structured and underestimation of species diversity occurs in the genus, which suggests a high degree of localised diversification and micro-endemism (Fei et al. 2012, Chen et al. 2018). In recent years, the approach of integrative taxonomy has made substantial progress with this species-rich group. However, only a short mitochondrial fragment of 16S gene was sequenced for many species in this genus and the phylogenetic relationships between most species in the genus have not been resolved (Ohler et al. 2011, Rowley et al. 2017b, Chen et al. 2018, Chen et al. 2023, Liu et al. 2023 etc.). This is most probably due to the insufficient phylogenetic information content in this kind of gene fragment (Chan et al. 2022).

In this study, two mitochondrial genes and one nuclear gene were amplified. On the 16S gene, the genetic distance between *Leptobrachelladushanensis* sp. nov. and its closely-related species *L.dong* was 1.6% which is at the same level with *L.bijie* and *L.jinyunensis*, but on COI gene, the genetic distance between them is 7.3%, much larger than that between other solid species (Suppl. materials 3, 4). Additionally, the haplotype network constructed, based on RAG1 gene sequences, also showed that all samples of *Leptobrachelladushanensis* sp. nov. shared one haplotype and there was no common haplotype between *Leptobrachelladushanensis* sp. nov. and its related species. This observation indicates a clear genetic differentiation between *Leptobrachelladushanensis* sp. nov. and its relatives, supporting its recognition as a distinct species. The lack of shared haplotypes further implies limited gene flow and potential geographic isolation between *Leptobrachelladushanensis* sp. nov. and its related species. The evidence for the interspecific divergence was further confirmed by the significant morphological differences. Accordingly, the results of molecular and morphological comparisons indicated the validation of these species.

## Supplementary Material

XML Treatment for Leptobrachella dushanensis

E81ACCDF-3F54-5409-B251-D4C618AC019B 10.3897/BDJ.12.e113427.suppl1 Supplementary material 1 Measurements Data type morphological Brief description Measurements of adult specimen of *Leptobrachelladushanensis* sp. nov. and *L.dong*. Units in mm. See abbreviations for characters in the Materials and Methods section. File: oo_957429.xlsx https://binary.pensoft.net/file/957429 Shize Li, Wei Li, Yanlin Cheng, Jing Liu, Gang Wei, Bin Wang

1D80AD3E-6470-5F36-8F54-E0809F64D77C 10.3897/BDJ.12.e113427.suppl2 Supplementary material 2 Diagnosis characters on morphology Data type morphological Brief description Diagnosis characters on morphology of *Leptobrachelladong* sp. nov. from other congeners. File: oo_957430.xlsx https://binary.pensoft.net/file/957430 Shize Li, Wei Li, Yanlin Cheng, Jing Liu, Gang Wei, Bin Wang

50E7B301-5D9C-5D9D-AD51-D65AAD1DEE0E 10.3897/BDJ.12.e113427.suppl3 Supplementary material 3 Uncorrected *p*-distance on the 16S rRNA gene Data type phylogenetic Brief description Uncorrected *p*-distance between *Leptobrachella* species on the 16S rRNA gene. Mean value of genetic distance is given in the lower half of the table. File: oo_957432.xlsx https://binary.pensoft.net/file/957432 Shize Li, Wei Li, Yanlin Cheng, Jing Liu, Gang Wei, Bin Wang

8307ABF8-F170-5103-8113-B09D5F269981 10.3897/BDJ.12.e113427.suppl4 Supplementary material 4 Uncorrected *p*-distance on the COI Data type phylogenetic Brief description Uncorrected *p*-distance between *Leptobrachella* species on the COI. Mean value of genetic distance is given in the lower half of the table. File: oo_957433.xlsx https://binary.pensoft.net/file/957433 Shize Li, Wei Li, Yanlin Cheng, Jing Liu, Gang Wei, Bin Wang

## Figures and Tables

**Table 1.** Information for samples used in molecular phylogenetic analyses in this study (/ = not available)

ID	Species	Locality	Voucher number	GenBank accession number		
				16S	COI	RAG1
1	*Leptobrachelladushanensis* sp. nov.	Dushan County, Guizhou Province, China	CIB DS20220409002	PP061389	PP061403	PP067958
2	*Leptobrachelladushanensis* sp. nov.	Dushan County, Guizhou Province, China	CIB DS20230310002	PP061391	PP061405	/
3	*Leptobrachelladushanensis* sp. nov.	Dushan County, Guizhou Province, China	CIB DS20230310001	PP061390	PP061404	/
4	*Leptobrachelladushanensis* sp. nov.	Dushan County, Guizhou Province, China	CIB DS20220409001	PP061388	PP061402	PP067957
5	*L.graminicola*	Mount Pu Ta Leng, Lao Cai, Vietnam	VNMN 010905	MZ224648	/	/
6	*L.graminicola*	Mount Pu Ta Leng, Lao Cai, Vietnam	VNMN 010908	MZ224653	/	/
7	*L.graminicola*	Mount Pu Ta Leng, Lao Cai, Vietnam	VNMN 010909	MZ224649	/	/
8	*L.graminicola*	Mount Pu Ta Leng, Lao Cai, Vietnam	TOLO2022.518	PP061392	PP061406	/
9	*L.graminicola*	Mount Pu Ta Leng, Lao Cai, Vietnam	VNMN 010904	MZ224651	/	/
10	*L.graminicola*	Mount Pu Ta Leng, Lao Cai, Vietnam	VNMN 010912	MZ224647	/	/
11	*L.graminicola*	Mount Pu Ta Leng, Lao Cai, Vietnam	VNMN 010910	MZ224655	/	/
12	*L.yeae*	Changshouqiao, Mount Emei, Sichuan, China	CIBEM1867	MT957005	/	MT975978
13	*L.yeae*	Linggongli, Mount Emei, Sichuan, China	CIBEMLGL19052104	MT957006	/	MT975979
14	*L.yeae*	Mount Emei, Sichuan, China	KIZ025778	KX811928	KX812166	/
15	*L.dong*	Congjiang County, Guizhou Province, China	CIB LB20220305005	OP764533	PP061396	/
16	*L.dong*	Congjiang County, Guizhou Province, China	CIB LB20220311002	OP764535	PP061398	OP776441
17	*L.dong*	Congjiang County, Guizhou Province, China	CIB LB20220306008	OP764534	PP061397	OP776439
18	*L.dong*	Suining County, Hunan Province, China	CIB ZNY2022001	OP764538	PP061399	OP776442
19	*L.dong*	Suining County, Hunan Province, China	CIB ZNY2022002	OP764539	PP061400	OP776443
20	*L.dong*	Tongdao County, Hunan Province, China	CIB WB2020277	OP764531	PP061401	OP776448
21	*L.bourreti*	Sapa, Lao Cai, Vietnam	1999.566	KR827860	/	/
22	*L.bourreti*	Sapa, Lao Cai Province, Vietnam	AMS R 177673	KR018124	/	/
23	*L.bourreti*	Ky Quan San, Lao Cai, Vietnam	AMS R.188515	MZ208835	/	/
24	*L.bourreti*	Mount Pu Ta Leng, Lao Cai, Vietnam	VNMN 010916	MZ209167	/	/
25	*L.bourreti*	Bat Xat District, Lao Cai, Vietnam	VNMN 010073	PP061393	PP061407	/
26	*L.bourreti*	Bat Xat District, Lao Cai, Vietnam	ZMMU-A5636-02280	MH055872	/	/
27	*L.jinyunensis*	Mt.Jinyun, Beibei District, Chongqing, China	CIB 119058	OQ024797	OQ024396	OQ031199
28	*L.jinyunensis*	Mt.Jinyun, Beibei District, Chongqing, China	CIB 119061	OQ024800	OQ024399	OQ031202
29	*L.chishuiensis*	Chishui National Nature Reserve, Chishui, Guizhou, China	CIBCS20190518047	MT117055	OQ024422	OQ031225
30	*L.chishuiensis*	Chishui National Nature Reserve, Chishui, Guizhou, China	CIBCS20190518049	MT117053	OQ024424	OQ031227
31	*L.bijie*	Mt. Baima, Qixingguan, Bijie, Guizhou, China	CIB 119068	OQ024807	OQ024406	OQ031208
32	*L.bijie*	Mt. Baima, Qixingguan, Bijie, Guizhou, China	CIB 119069	OQ024808	OQ024407	OQ031209
33	*L.jinshaensis*	Lengshuihe Nature Reserve, Jinsha County, Guizhou, China	CIBJS20200516005	OQ024816	OQ024417	OQ031220
34	*L.jinshaensis*	Lengshuihe Nature Reserve, Jinsha County, Guizhou, China	CIBJS20200516002	OQ024815	OQ024419	OQ031222
35	*L.suiyangensis*	Huoqiuba Nature Reserve, Suiyang County, Guizhou, China	GZNU20180606005	MK829649	/	OL800396
36	*L.suiyangensis*	Huoqiuba Nature Reserve, Suiyang County, Guizhou, China	GZNU20180606006	MK829650	/	OL800397
37	*L.purpuraventra*	Mt. Baima, Qixingguan, Bijie, Guizhou, China	CIB 119072	OQ024811	OQ024412	OQ031215
38	*L.purpuraventra*	Mt. Baima, Qixingguan, Bijie, Guizhou, China	CIB 119073	OQ024812	OQ024413	OQ031216
39	*L.niveimontis*	Daxueshan Nature Reserve, Yunnan Province, China	KIZ015734	MT302618	/	OL800394
40	*L.yunyangensis*	Qiyaoshan Nature Reserve, Yunyang County, Chongqing, China	GZNU20210622001	OL800364	/	OL800393
41	*L.eos*	Long Nai Khao, Phongsali, Laos	MNHN: 2004.0276	KR827862	KR087758	/
42	*L.eos*	Boun Tay, Phongsaly, Laos	NCSM 80551	MH055887	/	/
43	*L.purpura*	Yingjiang, Yunnan Province, China	SYS a006530	MG520354	/	/
44	*L.alpina*	Caiyanghe, Yunnan Province, China	KIZ049024	MH055867	/	/
45	*L.wulingensis*	Tianquanshan Forest Park, Zhangjiajie, Hunan Province, China	CSUFT 177	MT530315	/	/
46	*L.dorsospina*	Yushe Forest Park, Shuicheng County, Guizhou Province, China	SYS a004961	MW046194	/	/
47	*L.oshanensis*	Baoguosi, Mount Emei, Sichuan Province, China	CIBEMS20190421BGS1	MT957023	/	/
48	*L.oshanensis*	Shengshuige, Mount Emei, Sichuan, China	CIBEMS20190422SSG1-4	MT957025	/	/
49	*L.oshanensis*	Heilongjiang, Mount Emei, Sichuan, China	CIBEMS20190422HLJ1-4	MT957027	/	/
50	*L.oshanensis*	Shengshuige, Mount Emei, Sichuan, China	CIBEMS20190421SSG1-10	MT957031	/	/
51	*L.oshanensis*	Heilongjiang, Mount Emei, Sichuan, China	CIBEMS20190422HLJ1-2	MT957033	/	/
52	*L.korifi*	Doi Inthanon,Thailand	KUHE 19134	LC741033	/	/
53	*L.sinorensis*	Mae Hong Son,Thailand	KUHE 19809	LC741034	/	/
54	*L.murphyi*	Doi Inthanon, Chiang Mai, Thailand	KIZ034039	MZ710519	/	/
55	*L.tengchongensis*	Gaoligong Shan, Yunnan Province, China	SYS a004598	KU589209	/	/
56	*L.khasiorum*	Khasi Hills, Meghalaya, India	SDBDU 2009.329	KY022303	/	/
57	*L.tamdil*	Mizoram, India	MZMU2224	MW665130	/	/
58	*L.yingjiangensis*	Yingjiang County, Yunnan Province, China	SYS a006533	MG520350	/	/
59	*L.puhoatensis*	Pu Hu, Thanh Hoa, Vietnam	VNMN:2016 A.23	KY849587	/	/
60	*L.petrops*	Ba Vi National Park, Ha Tay, Vietnam	ROM 13483	MH055901	/	/
61	*L.namdongensis*	Thanh Hoa Province, Vietnam	VNUF A.2017.37	MK965389	/	/
62	*L.liui*	Wuyi Shan City, Fujian Province, China	SYS a001597	KM014547	/	/
63	*L.mangshanensis*	Mangshan, Hunan Province, China	MSZTC201701	MG132196	/	/
64	*L.verrucosa*	Lianshan Bijiashan Nature Reserve, Guangdong, China	GEP a059	OP279589	/	/
65	*L.shimentaina*	Shimentai Nature Reserve, Guangdong, China	SYS a004712	MH055926	/	/
66	*L.flaviglandulosa*	Xiaoqiaogou Nature Reserve, Yunnan Province, China	KIZ016072	MH055934	/	/
67	*L.yunkaiensis*	Dawuling Forest Station, Maoming City, Guangdong Province, China	SYS a004663	MH605584	/	/
68	*L.maoershanensis*	Mao’er Shan, Guangxi Province, China	KIZ07614	MH055927	/	/
69	*L.bashaensis*	Basha Nature Reserve, Congjiang County, Guizhou Province, China	GIB196403	MW136294	/	/
70	*L.laui*	Shenzhen City, Guangdong Province, China	SYS a002450	MH055904	/	/
71	*L.firthi*	Quang Nam Province, Vietnam	AMS R 171714	JQ739203	/	/
72	*L.isos*	Gia Lai, Vietnam	AMS R 176469	KT824767	/	/
73	*L.sungi*	Tam Dao, Vinh Phuc, Vietnam	ROM 20236	MH055858	/	/
74	*L.zhangyapingi*	Chiang Mai, Thailand	KIZ07258	MH055864	/	/
75	*L.pelodytoides*	Tam Dao, Vinh Phu, Vietnam	ROM18282	EF397244	/	/
76	*L.ventripunctata*	Wenlong, Yunnan Province, China	KIZ013621	MH055824	/	/
77	*L.feii*	Xiaoqiaogou Nature Reserve, Yunnan Province, China	KIZ048894	MT302634	/	/
78	*L.aspera*	Huanglianshan Nature Reserve, Lyuchun, Yunnan, China	SYS a007743	MW046199	/	/
79	*L.aerea*	Vilabuly, Savannakhet, Laos	NCSM 76038	MH055809	/	/
80	*L.minima*	Doi Phu Fa, Nan, Thailand	KIZ024317	MH055852	/	/
81	*L.nyx*	Ha Giang, Vietnam	ROM 36692	MH055816	/	/
82	*L.wuhuangmontis*	Pubei County, Guangxi Province, China	SYS a003485	MH605577	/	/
83	*L.shiwandashanensis*	Fangcheng City, Guangxi Province, China	NNU202103146	MZ326691	/	/
84	*L.wumingensis*	Wuming County, Guangxi, China	NNU 00283	OM935575	/	/
85	*L.pluvialis*	Fansipan, Lao Cai, Vietnam	ROM 30685	MH055843	/	/
86	*L.shangsiensis*	Guangxi Province, China	NHMG1401032	MK095460	/	/
87	*L.nahangensis*	Na Hang Nature Reserve, Tuyen Quang, Vietnam	ROM 7035	MH055853	/	/
88	*L.damingshanensis*	Guangxi Province., China	NNU202103281	MZ145229	/	/
89	*L.kalonensis*	Binh Thuan Province, Vietnam	AMNH A191762	KR018115	/	/
90	*L.bidoupensis*	Bidoup-Nui Ba National Park, Lam Dong, Vietnam	ZMMU-A-4797-01454	MH055945	/	/
91	*L.pallida*	Lam Dong, Vietnam	UNS00511	KU530190	/	/
92	*L.tadungensis*	Dak Nong Province, Vietnam	UNS00515	KR018121	/	/
93	*L.maculosa*	Ninh Thuan Province, Vietnam	AMS R 177660	KR018119	/	/
94	*L.pyrrhops*	Loc Bac, Lam Dong, Vietnam	ZMMU-A-4873-00158	MH055950	/	/
95	*L.macrops*	Phu Yen, Vietnam	ZMMU-A5823	MG787993	/	/
96	*L.rowleyae*	Da Nang City, Vietnam	ITBCZ2783	MG682552	/	/
97	*L.applebyi*	Phong Dien Nature Reserve, Thua Thien-Hue, Vietnam	KIZ010701	MH055947	/	/
98	*L.melica*	Cambodia, Ratanakiri	MVZ258198	HM133600	/	/
99	*L.ardens*	Kon Ka Kinh National Park, Gia Lai, Vietnam	ZMMU-NAP-06099	MH055949	/	/
100	*L.crocea*	Thua Thien-Hue, Vietnam	ZMMU-NAP-02274	MH055955	/	/
101	*L.tuberosa*	Kon Ka Kinh National Park, Gia Lai, Vietnam	ZMMU-NAP-02275	MH055959	/	/
102	*L.botsfordi*	Fansipan, Lao Cai, Vietnam	AMS R 176540	MH055952	/	/
103	*L.fuliginosa*	Phetchaburi, Thailand	KUHE 20197	LC201988	/	/
104	*L.melanoleuca*	Kapoe, Ranong, Thailand	KIZ018031	MH055967	/	/
105	*L.neangi*	Veal Veng District, Pursat Province, Cambodia	CBC 1624	MT644613	/	/
106	*L.brevicrus*	Gunung Mulu National Park, Sarawak, Malaysia	UNIMAS 8957	KJ831303	/	/
107	*L.itiokai*	Mulu National Park, Sarawak, Malaysia	KUHE:55897	LC137805	/	/
108	*L.parva*	Mulu National Park, Sarawak, Malaysia	KUHE:55308	LC056791	/	/
109	*L.baluensis*	Tambunan, Sabah, Borneo, Malaysia	SP 21604	LC056792	/	/
110	*L.mjobergi*	Gading NP, Sarawak, Borneo, Malaysia	KUHE:47872	LC056787	/	/
111	*L.juliandringi*	Mulu NP, Sarawak, Borneo, Malaysia	KUHE 55333	LC056780	/	/
112	*L.sabahmontana*	Borneo, Malaysia	BORNEENSIS 12632	AB847551	/	/
113	*L.dringi*	Gunung Mulu, Malaysia	KUHE:55610	AB847553	/	/
114	*L.picta*	Borneo, Malaysia	UNIMAS 8705	KJ831295	/	/
115	*L.fritinniens*	Danum Valley Field Center, Sabah, Malaysia	FMNH 244800	MH055971	/	/
116	*L.arayai*	Liwagu, Kinabalu, Malaysia	BORNEEISIS 22931	AB847558	/	/
117	*L.hamidi*	Bukit Lanjan, Selangor, Malaysia	KUHE17545	AB969286	/	/
118	*L.marmorata*	Borneo, Malaysia	KUHE53227	AB969289	/	/
119	*L.gracilis*	Bukit Kana, Sarawak, Malaysia	FMNH 273682	MH055972	/	/
120	*L.maura*	Borneo, Malaysia	SP21450	AB847559	/	/
121	*L.heteropus*	Larut, Perak, Malaysia	KUHE15487	AB530453	/	/
122	*L.sola*	Gunung Stong, Kelantan, Malaysia	KU RMB20973	MH055973	/	/
123	*L.kecil*	Cameron, Malaysia	KUHE 52440	LC202004	/	/
124	*L.kajangensis*	Tioman, Malaysia	LSUHC 4431	LC202001	/	/
125	*Megophrysglandulosa*	Yunnan Province, China	KIZ048439	KX811762	KX812165	/
126	*Leptobrachiumhuashen*	Yunnan Province, China	KIZ049025	KX811931	KX812075	/

**Table 2.** References for morphological characters for congeners of the genus *Leptobrachella*.

ID	Species	References
1	*L.aerea* (Rowley, Stuart, Richards, Phimmachak & Sivongxay, 2010)	Rowley et al. 2010a
2	*L.alpina* (Fei, Ye & Li, 1990)	Fei et al. 1990
3	*L.applebyi* (Rowley & Cao, 2009)	Rowley and Cao 2009
4	*L.arayai* (Matsui, 1997)	Matsui 1997
5	*L.ardens* (Rowley, Tran, Le, Dau, Peloso, Nguyen, Hoang, Nguyen & Ziegler, 2016)	Rowley et al. 2016
6	*L.aspera* Wang, Lyu, Qi & Wang, 2020	Wang et al. 2020
7	*L.baluensis* Smith, 1931	Smith 1931
8	*L.bashaensis* Lyu, Dai, Wei, He, Yuan, Shi, Zhou, Ran, Kuang, Guo, Wei & Yuan, 2020	Lyu et al. 2020
9	*L.bidoupensis* (Rowley, Le, Tran & Hoang, 2011)	Rowley et al. 2011
10	*L.bijie* Wang, Li, Li, Chen & Wang, 2019	Wang et al. 2019
11	*L.bondangensis* Eto, Matsui, Hamidy, Munir & Isk &ar, 2018	Eto et al. 2018
12	*L.botsfordi* (Rowley, Dau & Nguyen, 2013)	Rowley et al. 2013
13	*L.bourreti* (Dubois, 1983)	Dubois 1983
14	*L.brevicrus* Dring, 1983	Dring 1983, Eto et al. 2015
15	*L.chishuiensis* Li, Liu, Wei & Wang, 2020	Li et al. 2020
16	*L.crocea* (Rowley, Hoang, Le, Dau & Cao, 2010)	Rowley et al. 2010b
17	*L.damingshanensis* Chen, Yu, Cheng, Meng, Wei, Zhou, Lu, 2021	Chen et al. 2021b
18	*L.dorsospina* Wang, Lyu, Qi & Wang, 2020	Wang et al. 2020
19	*L.dringi* (Dubois, 1987)	Dubois 1987
20	*L.dong* Liu, Shi, Li, Zhang, Xiang, Wei, and Wang, 2023	Liu et al. 2023
21	*L.eos* (Ohler, Wollenberg, Grosjean, Hendrix, Vences, Ziegler & Dubois, 2011)	Ohler et al. 2011
22	*L.feii* Chen, Yuan & Che, 2020	Chen et al. 2020
23	*L.firthi* (Rowley, Hoang, Dau, Le & Cao, 2012)	Rowley et al. 2012
24	*L.flaviglandulosa* Chen, Wang & Che, 2020	Chen et al. 2020
25	*L.fritinniens* (Dehling & Matsui, 2013)	Dehling and Matsui 2013
26	*L.fuliginosa* (Matsui, 2006)	Matsui 2006
27	*L.fusca* Eto, Matsui, Hamidy, Munir & Isk &ar, 2018	Eto et al. 2018
28	*L.gracilis* (Günther, 1872)	Dehling 2012a
29	*L.graminicola* Nguyen, Tapley, Nguyen, Luong & Rowley, 2021	Nguyen et al. 2021
30	*L.hamidi* (Matsui, 1997)	Matsui 1997
31	*L.heteropus* (Boulenger, 1900)	Boulenger 1900
32	*L.isos* (Rowley, Stuart, Neang, Hoang, Dau, Nguyen & Emmett, 2015)	Rowley et al. 2015
33	*L.itiokai* Eto, Matsui & Nishikawa, 2016	Eto et al. 2016
34	*L.jinshaensis* Cheng, Shi, Li, Liu, Li & Wang, 2021	Cheng et al. 2021
35	*L.jinyunensis* Shi, Shen, Wang, Jiang & Wang, 2023	Shi et al. 2023
36	*L.juliandringi* Eto, Matsui & Nishikawa, 2015	Eto et al. 2015
37	*L.kajangensis* (Grismer, Grismer & Youmans, 2004)	Grismer et al. 2004
38	*L.kalonensis* (Rowley, Tran, Le, Dau, Peloso, Nguyen, Hoang, Nguyen & Ziegler, 2016)	Rowley et al. 2016
39	*L.kecil* (Matsui, Belabut, Ahmad & Yong, 2009)	Matsui et al. 2009
40	*L.khasiorum* (Das, Tron, Rangad & Hooroo, 2010)	Das et al. 2010
41	*L.korifi* Matsui, Panha & Eto, 2023	Matsui et al. 2023
42	*L.lateralis* (Anderson, 1871)	Anderson 1871, Humtsoe et al. 2008
43	*L.laui* (Sung, Yang & Wang, 2014)	Sung et al. 2014
44	*L.liui* (Fei & Ye, 1990)	Fei et al. 1990, Sung et al. 2014
45	*L.macrops* (Duong, Do, Ngo, Nguyen & Poyarkov, 2018)	Duong et al. 2018
46	*L.maculosa* (Rowley, Tran, Le, Dau, Peloso, Nguyen, Hoang, Nguyen & Ziegler, 2016)	Rowley et al. 2016
47	*L.mangshanensis* (Hou, Zhang, Hu, Li, Shi, Chen, Mo & Wang, 2018)	Hou et al. 2018
48	*L.maoershanensis* (Yuan, Sun, Chen, Rowley & Che, 2017)	Yuan et al. 2017
49	*L.marmorata* (Matsui, Zainudin & Nishikawa, 2014)	Matsui et al. 2014b
50	*L.maura* (Inger, Lakim, Biun & Yambun, 1997)	Inger et al. 1997
51	*L.melanoleuca* (Matsui, 2006)	Matsui 2006
52	*L.melica* (Rowley, Stuart, Neang & Emmett, 2010)	Rowley et al. 2010a
53	*L.minima* (Taylor, 1962)	Ohler et al. 2011, Taylor 1962
54	*L.mjobergi* Smith, 1925	Smith 1925
55	*L.murphyi* Chen, Suwannapoom, Wu, Poyarkov, Xu, Pawangkhanant & Che, 2021	Chen et al. 2021
56	*L.nahangensis* (Lathrop, Murphy, Orlov & Ho, 1998)	Lathrop et al. 1998
57	*L.namdongensis* Hoang, Nguyen, Luu, Nguyen & Jiang, 2019	Hoang et al. 2019
58	*L.natunae* (Günther, 1895)	Günther 1895
59	*L.neangi* Stuart & Rowley, 2020	Stuart and Rowley 2020
60	*L.niveimontis* Chen, Poyarkov, Yuan & Che, 2020	Chen et al. 2020
61	*L.nokrekensis* (Mathew & Sen, 2010)	Mathew and Sen 2010
62	*L.nyx* (Ohler, Wollenberg, Grosjean, Hendrix, Vences, Ziegler & Dubois, 2011)	Ohler et al. 2011
63	*L.oshanensis* (Liu, 1950)	Liu 1950, Shi et al. 2023
64	*L.pallida* (Rowley, Tran, Le, Dau, Peloso, Nguyen, Hoang, Nguyen &	Rowley et al. 2016
65	*L.palmata* Inger & Stuebing, 1992	Inger and Stuebing 1992
66	*L.parva* Dring, 1983	Dring 1983
67	*L.pelodytoides* (Boulenger, 1893)	Boulenger 1893, Ohler et al. 2011
68	*L.petrops* (Rowley, Dau, Hoang, Le, Cutajar & Nguyen, 2017)	Rowley et al. 2017b
69	*L.phiadenensis* Luong, Hoang, Pham, Ziegler, and Nguyen, 2023	Luong et al. 2023
70	*L.phiaoacensis*Luong, Hoang, Pham, Ziegler, and Nguyen, 2023	Luong et al. 2023
71	*L.picta* (Malkmus, 1992)	Malkmus 1992
72	*L.pingbianensis* (Rao, Hui, Zhu & Ma, 2022 "2020")	Zhu and Rao 2020
73	*L.platycephala* (Dehling, 2012)	Dehling 2012b
74	*L.pluvialis* (Ohler, Marquis, Swan & Grosjean, 2000)	Ohler et al. 2000
75	*L.puhoatensis* (Rowley, Dau & Cao, 2017)	Rowley et al. 2017a
76	*L.purpuraventra* Wang, Li, Li, Chen & Wang, 2019	Wang et al. 2019
77	*L.purpurus* (Yang, Zeng & Wang, 2018)	Yang et al. 2018
78	*L.pyrrhops* (Poyarkov, Rowley, Gogoleva, Vassilieva, Galoyan & Orlov, 2015)	Poyarkov et al. 2015
79	*L.rowleyae* (Nguyen, Poyarkov, Le, Vo, Ninh, Duong, Murphy & Sang, 2018)	Nguyen et al. 2018
80	*L.sabahmontana* (Matsui, Nishikawa & Yambun, 2014)	Matsui et al. 2014a
81	*L.serasanae* Dring, 1983	Dring 1983
82	*L.shangsiensis* Chen, Liao, Zhou & Mo, 2019	Chen et al. 2019
83	*L.shiwanshanensis* Chen, Peng, Pan, Liao, Liu & Huang, 2021	Chen et al. 2021a
84	*L.shimentaina* Wang, Lyu & Wang, 2022	Wang et al. 2022
85	*L.sinorensis* Matsui, Panha & Eto, 2023	Matsui et al. 2023
86	*L.sola* (Matsui, 2006)	Matsui 2006
87	*L.suiyangensis* Luo, Xiao, Gao & Zhou, 2020	Luo et al. 2020
88	*L.sungi* (Lathrop, Murphy, Orlov & Ho, 1998)	Lathrop et al. 1998
89	*L.tadungensis* (Rowley, Tran, Le, Dau, Peloso, Nguyen, Hoang, Nguyen & Ziegler, 2016)	Rowley et al. 2016
90	*L.tamdil* (Sengupta, Sailo, Lalremsanga, Das & Das, 2010)	Sengupta et al. 2010
91	*L.tengchongensis* (Yang, Wang, Chen & Rao, 2016)	Yang et al. 2018
92	*L.tuberosa* (Inger, Orlov & Darevsky, 1999)	Inger et al. 1999
93	*L.ventripunctata* (Fei, Ye & Li, 1990)	Fei et al. 1990
94	*L.verrucosa* Wang, Zeng, Lin & Li, 2022	Lin et al. 2022
95	*L.wuhuangmontis* Wang, Yang & Wang, 2018	Wang et al. 2018
96	*L.wulingensis* Qian, Xiao, Cao, Xiao & Yang, 2020	Qian et al. 2020
97	*L.wumingensis* Chen, Peng, Li, and Yu, 2023	Chen et al. 2023
98	*L.yeae* Shi, Hou, Song, Jiang & Wang, 2021	Shi et al. 2021
99	*L.yingjiangensis* (Yang, Zeng & Wang, 2018)	Yang et al. 2018
100	*L.yunyangensis* Luo, Deng & Zhou, 2022	Luo et al. 2022
101	*L.yunkaiensis* Wang, Li, Lyu & Wang, 2018	Wang et al. 2018
102	*L.zhangyapingi* (Jiang, Yan, Suwannapoom, Chomdej & Che, 2013)	Jiang et al. 2013

**Table 3.** Measurements of *Leptobrachelladushanensis* sp. nov and *L.dong* from Guizhou and Hunan populations. Units in mm. In addition, the results of one-way ANOVA with *p*-values for morphometric comparisons between *Leptobrachelladushanensis* sp. nov. and *L.dong* from Guizhou and Hunan populations (* *p* < 0.05, ** *p* < 0.01) are given. See abbreviations for characters in the Materials and Methods section.

Character	*Leptobrachelladushanensis* sp.nov. (n = 4)		*L.dong* (n = 4) Guizhou		*L.dong* (n = 11) Hunan		*p*-value	
	Ranging	Mean ± SD	Ranging	Mean ± SD	Ranging	Mean ± SD	Guizhou	Hunan
SVL	31.9 – 32.9	32.3 ± 0.4	29.5 – 31.2	30.4 ± 0.86	29.2 – 34.2	31.1 ± 1.4	0.006**	0.169
HDL	10.5 – 10.8	10.6 ± 0.1	9.6 – 10.8	10.1 ± 0.5	10.0 – 11.7	10.7 ± 0.5	0.694	0.025*
HDW	10.0 – 10.4	10.1 ± 0.2	9.8 – 10.9	10.5 ± 0.5	10.5 – 12.2	11.2 ± 0.6	0.001**	0.000**
SL	4.2 – 4.4	4.2 ± 0.1	4.2 – 4.9	4.6 ± 0.3	4.0 – 5.0	4.4 ± 0.3	0.027*	0.044*
IND	2.6 – 2.7	2.6 ± 0.1	3.5 – 4.4	3.8 ± 0.4	2.8 – 3.6	3.1 ± 0.3	0.000**	0.000**
IOD	2.8 – 3.1	2.9 ± 0.1	2.3 – 3.2	2.8 ± 0.4	2.8 – 3.9	3.4 ± 0.4	0.788	0.001**
UEW	2.9 – 3.1	3.0 ± 0.1	2.8 – 3.4	3.1 ± 0.3	2.6 – 3.1	2.9 ± 0.2	0.094	0.946
ED	3.8 – 4.0	3.9 ± 0.1	3.9 – 4.4	4.1 ± 0.2	3.6 – 4.4	4.2 ± 0.2	0.039*	0.006**
TYD	1.6 – 1.8	1.7 ± 0.1	1.9 – 2.2	2.1 ± 0.1	1.5 – 1.7	1.6 ± 0.1	0.001**	0.705
LAL	14.4 – 14.5	14.5 ± 0.1	13.5 – 14.2	13.9 ± 0.3	14.3 – 15.5	14.8 ± 0.4	0.127	0.011*
ML	8.0 – 8.5	8.1 ± 0.2	7.3 – 8.3	7.9 ± 0.4	7.2 – 8.6	7.9 ± 0.4	0.216	0.750
TL	15.9 – 16.0	15.9 ± 0.1	14.5 – 14.7	14.6 ± 0.1	14.5 – 16.4	15.5 ± 0.6	0.079	0.749
FL	14.8 – 15.0	14.9 ± 0.1	13.5 – 15	14.2 ± 0.6	13.5 – 15.5	15.0 ± 0.6	0.625	0.408
HLL	50.0 – 50.5	50.3 ± 0.3	41.3 – 44.2	43.3 ± 1.3	46.2 – 51.6	49.1 ± 1.6	0.018*	0.564
